# Altered structural brain networks in linguistic variants of frontotemporal dementia

**DOI:** 10.1007/s11682-021-00560-2

**Published:** 2021-11-10

**Authors:** Salvatore Nigro, Benedetta Tafuri, Daniele Urso, Roberto De Blasi, Alessia Cedola, Giuseppe Gigli, Giancarlo Logroscino, for the Frontotemporal Lobar Degeneration Neuroimaging Initiative*

**Affiliations:** 1 Institute of Nanotechnology (NANOTEC), National Research Council, Lecce, Italy; 2 Center for Neurodegenerative Diseases and the Aging Brain, Department of Clinical Research in Neurology, University of Bari Aldo Moro, Pia Fondazione Cardinale G. Panico, Tricase, Lecce, Italy; 3 Department of Basic Medicine, Neuroscience, and Sense Organs, University of Bari Aldo Moro, Bari, Italy; 4 Department of Neurosciences, Institute of Psychiatry, Psychology and Neuroscience, King’s College London, De Crespigny Park, London, SE5 8AF UK; 5 Department of Radiology, Pia Fondazione Cardinale G. Panico, Tricase, Lecce, Italy; 6 Department of Mathematics and Physics Ennio De Giorgi, University of Salento, Campus Ecotekne, Lecce, Italy

**Keywords:** Semantic variant of primary progressive aphasia, Nonfluent variant of primary progressive aphasia, Graph analysis, Gray matter structural covariance networks, Brain networks

## Abstract

Semantic (svPPA) and nonfluent (nfvPPA) variants of primary progressive aphasia (PPA) have recently been associated with distinct patterns of white matter and functional network alterations in left frontoinsular and anterior temporal regions, respectively. Little information exists, however, about the topological characteristics of gray matter covariance networks in these two PPA variants. In the present study, we used a graph theory approach to describe the structural covariance network organization in 34 patients with svPPA, 34 patients with nfvPPA and 110 healthy controls. All participants underwent a 3 T structural MRI. Next, we used cortical thickness values and subcortical volumes to define subject-specific connectivity networks. Patients with svPPA and nfvPPA were characterized by higher values of normalized characteristic path length compared with controls. Moreover, svPPA patients had lower values of normalized clustering coefficient relative to healthy controls. At a regional level, patients with svPPA showed a reduced connectivity and impaired information processing in temporal and limbic brain areas relative to controls and nfvPPA patients. By contrast, local network changes in patients with nfvPPA were focused on frontal brain regions such as the pars opercularis and the middle frontal cortex. Of note, a predominance of local metric changes was observed in the left hemisphere in both nfvPPA and svPPA brain networks. Taken together, these findings provide new evidences of a suboptimal topological organization of the structural covariance networks in svPPA and nfvPPA patients. Moreover, we further confirm that distinct patterns of structural network alterations are related to neurodegenerative mechanisms underlying each PPA variant.

**Supplementary Information:**

The online version contains supplementary material available at 10.1007/s11682-021-00560-2.

## Introduction

Frontotemporal dementia (FTD) is a heterogeneous group of neurodegenerative disorders characterized by behavioral disturbances, impairment of executive functions or language deficits (Bang et al., 2015; Coyle-Gilchrist et al., 2016). Based on the presence of prominent speech and language deteriorations, two main clinical FTD variants, namely the nonfluent variant of primary progressive aphasia (nfvPPA) and the semantic variant of PPA (svPPA) can be identified (Gorno-Tempini et al., 2011; Leyton et al., 2011; Tee & Gorno-Tempini, 2019). In general, individuals with svPPA show difficulty in naming and objects/faces identification (Gorno-Tempini et al., 2004, 2011). Further studies also reported surface dyslexia and spared speech production (Gorno-Tempini et al., 2011; Snowden et al., 2011). On the other hand, nfvPPA patients are characterized by the presence of agrammatism and effort toward spontaneous speech (Gorno-Tempini et al., 2011). Patients with nfvPPA also showed impaired prosody as well as phonetic errors (Ash et al., 2010; Wilson et al., 2010).

Over the past decade, several neuroimaging studies have investigated structural and functional brain abnormalities related to language impairment in patients with svPPA and nfvPPA (Galantucci et al., 2011; Montembeault et al., 2018; Ranasinghe et al., 2017; Tee & Gorno-Tempini, 2019). In svPPA patients, gray matter atrophy has been reported in the anterior temporal lobes, insula, amygdala and hippocampus (Brambati et al., 2009; Collins et al., 2017). Agrammatic symptoms typical reported in nfvPPA patients have been associated with gray matter decrease in several cortical and subcortical brain regions such as the inferior frontal gyrus, insula, supplementary motor cortex and striatum (Mandelli, Vitali, et al., 2016a, 2016b). Language impairments in svPPA and nfvPPA patients have also been associated with hypoperfusion and functional connectivity decrease in the left temporal lobe and inferior-frontal gyrus, respectively (Josephs et al., 2018; Ranasinghe et al., 2017). In more recent years, few studies have combined MRI data and graph analysis to assess selective disruptions of the large-scale network associated with PPA (Mandelli et al., 2018; Mandelli et al., 2016a, 2016b; P. Reyes et al., 2018; P. A. Reyes et al., 2019). In this context, by diffusion tensor imaging (DTI) and functional MRI (fMRI) data, networks analysis investigations have reported altered patterns of white matter and functional connectivity in temporal and occipital lobes of patients with svPPA in comparison to healthy controls (P. Reyes et al., 2018; P. A. Reyes et al., 2019). By contrast, network disconnections were observed in frontal brain regions of patients with nfvPPA (Mandelli et al., 2018; P. Reyes et al., 2018; P. A. Reyes et al., 2019).

Despite recent progress in understanding structural and functional brain network alterations in patients with PPA, to date no study has used the inter-regional gray matter covariation to describe brain network changes in linguistic variants of FTD. However, structural covariance analysis has proved to be a robust approach to investigate the brain network organization in several neurological conditions (Pereira et al., 2015; Yao et al., 2010; Yun et al., 2020). Moreover, gray matter covariance networks construction has proved to be less sensitive to noise in comparison to that of functional and DTI-based networks (Bruno et al., 2017; Hosseini et al., 2016), requiring relatively lower computational loads (Yao et al., 2010). In a recent study, we have also demonstrated the usefulness of inter-regional gray matter covariation to assess connectivity abnormalities in patients with behavioral variant of FTD in comparison to healthy controls (Nigro et al., 2021).

The aim of the current study was to characterize the structural covariance brain network organization in linguistic variant of FTD. To this end, we used cortical thickness values and subcortical volumes to define subject-specific anatomical connectivity. Then, we investigated segregation and integration abilities within brain networks at both global and local level. We hypothesized that structural covariance networks of PPA patients would show global network changes in comparison to healthy controls. We also expected distinct patterns of structural disconnection in svPPA and nfvPPA patients with prominent alterations in temporal and frontal brain regions, respectively.

## Materials and method

### Patients

Data used in current study were obtained from the Frontotemporal Lobar Degeneration Neuroimaging Initiative (FTLDNI) database. The FTLDNI was funded through the National Institute of Aging and started in 2010. The primary goals of FTLDNI are to identify neuroimaging modalities and methods of analysis for tracking frontotemporal lobar degeneration and to assess the value of imaging versus other biomarkers in diagnostic roles. The project is the result of collaborative efforts at three different sites in North America. For up-to-date information on participation and protocol, please visit: http://memory.ucsf.edu/research. Data was downloaded through the LONI platform after approved by the data access committee. We included 110 HC and 68 patients with PPA (34 nfvPPA and 34 svPPA) who had a valid baseline T1-weighted MR images. Demographic information and neuropsychological data are shown in Table 1. Of note, we considered only participants scanned at University of California, San Francisco (UCSF), the largest recruiting center, in order to avoid potential bias due to different scanners and acquisition imaging protocol. Approval for the FTLDNI protocol has been granted by institutional review board at the study site.

**Table 1:** Demographic, clinical, and neuroimaging data of sample

	HC (n = 110)	svPPA (n = 34)	nfvPPA (n = 34)
Demographic and clinical data			
Age at exam (years)^a^	63.12 ± 7.49	62.91 ± 6.29	68.32 ± 7.27
Gender (M/F)	49/61	20/14	15/19
Education (years)	17.50 ± 1.92	16.94 ± 3.09	16.51 ± 3.39
MMSE^b^	29.35 ± 0.77	24.97 ± 5.10	25.54 ± 4.04
CDR Total score	–	0.63 ± 0.31	0.48 ± 0.40
CDR-SOB score^c^	–	3.40 ± 1.72	2.15 ± 2.11
Semantic Fluency (animal)^b^	24.25 ± 5.53	9.03 ± 4.15	10.72 ± 6.01
Lexical Fluency (phonemic)^b,c^	16.37 ± 4.34	8.97 ± 4.47	5.53 ± 3.35
BNT (max = 15)^b,c^	14.46 ± 0.78	5.90 ± 3.42	12.64 ± 2.28
Neuroimaging data			
Intracanial Volume (ml)	1517.69 ± 69.04	1535.34 ± 156.76	1471.09 ± 171.17

*MMSE*: Mini-Mental State Examination; *CDR Total:* Clinical Dementia Rating Total score; *CDR-SOB*: Clinical Dementia Rating: Box Score; *BNT*: Boston Naming Test; *HC:* healthy controls; *svPPA:* patients with semantic variant of primary progressive aphasia; *nfvPPA:* patients with nonfluent variant of primary progressive aphasia

^a^p < 0.05 nfvPPA patients versus controls and svPPA patients

^b^p < 0.05 svPPA and nfvPPA patients versus controls

^c^p < 0.05 svPPA patients versus nfvPPA patients

### MRI acquisition and processing

MR images were acquired on a 3 T Siemens Trio Tim system equipped with a 12-channel head coil at the UCSF Neuroscience Imaging Center. Structural images were acquired using a T1 weighted MPRAGE (TR/TE = 2,300/2.9 ms, matrix = 240 × 256 × 160, isotropic voxels 1 mm3, slice thickness = 1 mm). T1-weighted images were analyzed using FreeSurfer (version 6.0) (http://www.nmr.mgh.harvard.edu/martinos) to compute morphological properties for cortical and subcortical brain regions (Dale et al., 1999; Fischl & Dale, 2000; Fischl et al., 1999, 2004). All images were checked for reconstruction cortical surface errors.

### Gray matter network construction

For each subject, Desikan-Killianny atlas was used to extract average cortical thickness values in 68 cortical brain regions (Desikan et al., 2006). A complete list of cortical areas is provided in Supplementary Materials (Table S1). Volumetric values of the putamen, caudate, thalamus, pallidum, hippocampus, accumbens and amygdala for each hemisphere were also computed (Dale et al., 1999; Fischl et al., 2004). Next, a linear regression analysis was performed at every region to remove the effects of age, gender and total intracranial volume. Then, the residuals of this regression were z-score transformed using mean and standard deviation values of each brain region calculated in the control group (Li et al., 2020; Yun et al., 2016, 2020). Finally, the structural connectivity value (network edge weight) between each pair of regions was calculated using the following measure of joint variation (Yun et al., 2016, 2020) distributed between 0 and 1:

$${{\varvec{J}}{\varvec{o}}{\varvec{i}}{\varvec{n}}{\varvec{t}} {\varvec{v}}{\varvec{a}}{\varvec{r}}{\varvec{i}}{\varvec{a}}{\varvec{t}}{\varvec{i}}{\varvec{o}}{\varvec{n}} }_{{\varvec{b}}{\varvec{e}}{\varvec{t}}{\varvec{w}}{\varvec{e}}{\varvec{e}}{\varvec{n}} {\varvec{t}}{\varvec{h}}{\varvec{e}} {\varvec{i}}{\varvec{t}}{\varvec{h}} \left({\varvec{f}}{\varvec{o}}{\varvec{r}} {\varvec{i}}=1 {\varvec{t}}{\varvec{o}} 82\right){\varvec{a}}{\varvec{n}}{\varvec{d}} {\varvec{j}}{\varvec{t}}{\varvec{h}}({\varvec{f}}{\varvec{o}}{\varvec{r}} {\varvec{j}}=1 {\varvec{t}}{\varvec{o}} 82) {\varvec{r}}{\varvec{e}}{\varvec{g}}{\varvec{i}}{\varvec{o}}{\varvec{n}}{\varvec{s}} {\varvec{o}}{\varvec{f}} {\varvec{i}}{\varvec{n}}{\varvec{t}}{\varvec{e}}{\varvec{r}}{\varvec{e}}{\varvec{s}}{\varvec{t}}}$$ =

*1/exp{[(z-score value of ith region of interest)—(z-score value of jth region of interest)]*^*^2*^*}* (1)

### Graph theory analysis

Topological organization of structural covariance networks were assessed using global and local graph metrics. At a global level, the normalized clustering coefficient (γ) and the normalized characteristic path length (λ) were calculated to investigate the degree of segregation and integration of the network (-). Next, the small-worldness index (σ) was computed as the ratio of gamma to lambda. Values of sigma higher than 1.1 are indicative of a small-world network configuration suggesting an efficient information processing in the human brain networks at low wiring costs (Achard & Bullmore, 2007; Watts & Strogatz, 1998). Integration and segregation properties were also quantified at a regional level using the local efficiency and clustering coefficient, respectively (Rubinov & Sporns, 2010; Watts & Strogatz, 1998; Zuo et al., 2012). In particular, local efficiency was used to measure the ability of information transfer between a node and the remaining nodes in the network (Rubinov & Sporns, 2010). Clustering coefficient allowed to quantify the propensity of a node to communicate with its immediate neighborhood (Rubinov & Sporns, 2010; Watts & Strogatz, 1998). Nodal degree was also evaluated to quantify the centrality of each node within the network (Zuo et al., 2012). All graph measures were evaluated across a range of network densities ranging from 10 to 40%. This range of thresholds allowed to preserve a small-world configuration in each individual network minimizing the number of spurious edges (Achard & Bullmore, 2007; He et al., 2007). Graph theoretical Network Analysis (GRETNA) package was used to calculate all network properties (Wang et al., 2015).

### Statistical analysis

Demographic, neuroimaging and neuropsychological variables were compared between groups using Wilcoxon-Mann–Whitney test. The difference in sex distribution among groups was evaluated using Chi-square test.

To assess group differences in network properties, the area under the curve (AUC) for each network measure was calculated. Specifically, we calculated both global and local metrics at each density. Next, the AUC was computed by integrating the curve over density range, providing a scalar feature for the topological property not depending on specific threshold selection. Nonparametric permutation testing (5000 repetitions) was then conducted to test the statistical significance differences between the PPA patients and control group. The top 20 ranked brain regions were also identified according to each local graph metrics in controls, svPPA and nfvPPA patients. The relationships between network metrics and clinical data of patients with svPPA and nfvPPA were also tested using the Spearman correlation (p-value < 0.05). A Bonferroni correction procedure was employed in all statistical analyses to correct for multiple comparisons. The critical statistical threshold was set to p < 0.05. The Hedge's g statistic was also used to measure the effect size for the difference in local and global metrics between patients and healthy controls (Hedges & Olkin, 1985). Of note, differences in global and local metrics between PPA groups were also evaluated including the clinical dementia rating as a nuisance variable. The BrainNet viewer (http://www.nitrc.org/projects/bnv/) was used to visualize significant regional differences between groups (Xia et al., 2013).

## Results

### Demographic and clinical characteristics

No significant differences in sex, years of education, and intracranial volume were found between PPA patients and healthy controls (p > 0.05). However, patients with nfvPPA had a significantly older age compared with healthy control participants and svPPA patients (p-value < 0.05) (Table 1). PPA patients showed also reduced MMSE values when compared to healthy controls, although nfvPPA and svPPA patients had similar MMSE values and CDR (Clinical Dementia Rating Scale) scores.

### Global network properties

Small-world network configuration was observed in structural covariance networks of controls and PPA patients (1.29 < σ_HC_ < 2.57; 1.15 < σ_svPPA_ < 2.19; 1.20 < σ_nfvPPA_ < 2.56). However, the small-worldness index was significant smaller in svPPA patients than nfvPPA patients and controls. Moreover, the normalized characteristic path length (λ) values in both svPPA and nfvPPA patients were greater than those of controls (Table 2, p-value < 0.05, Bonferroni corrected). Compared with the control participants and nfvPPA patients, svPPA group exhibited also significantly less normalized clustering coefficient (γ) ( (Table 2, p-value < 0.05, Bonferroni corrected). No significant correlations were found between clinical variables and global network measures.

**Table 2:** Main effect of group in the global network metrics

	HC	svPPA	nfvPPA	HC vs svPPA p-value (effect size)	HC vs nfvPPA p-value (effect size)	svPPA vs nfvPPA p-value (effect size)
σ	0.52 ± 0.07	0.44 ± 0.05	0.51 ± 0.07	< 0.001 (1.34)	0.42 (0.16)	< 0.001 (−1.16)
λ	0.42 ± 0.01	0.44 ± 0.02	0.43 ± 0.02	< 0.001 (−1.78)	< 0.001 (−0.66)	0.001 (0.83)
γ	0.75 ± 0.09	0.67 ± 0.07	0.75 ± 0.09	< 0.001 (0.98)	0.95 (−0.01)	< 0.001 (−0.98)

All graph measure values are expressed as the area under the curve (AUC) across density range

σ, small-worldness index; λ, normalized characteristic path length; γ, normalized clustering coefficient; *Eglob:* global efficiency; *HC:* healthy controls; *svPPA:* semantic variant of primary progressive aphasia; *nfvPPA:* progressive nonfluent variant of primary progressive aphasia

### Local network properties

**Fig. 1:**
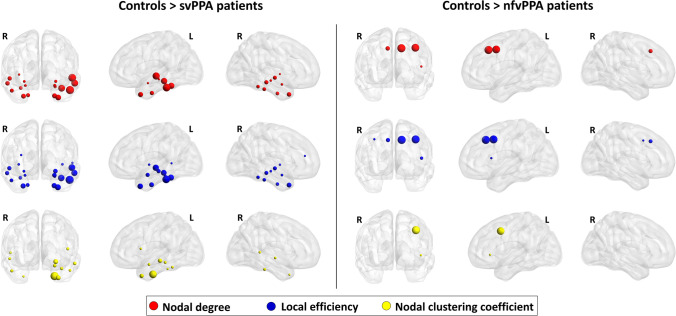
Left panel: cortical and subcortical brain regions showing reduced local properties between patients with semantic variant of primary progressive aphasia (svPPA) and controls (p < 0.05, Bonferroni corrected); Right panel:cortical and subcortical brain regions showing reduced local properties between patients with nonfluent variant of primary progressive aphasia (nfvPPA) and controls (p < 0.05, Bonferroni corrected). Node size is proportional to the effect size of the difference between patients and controls in local graph meausure

**Fig. 2:**
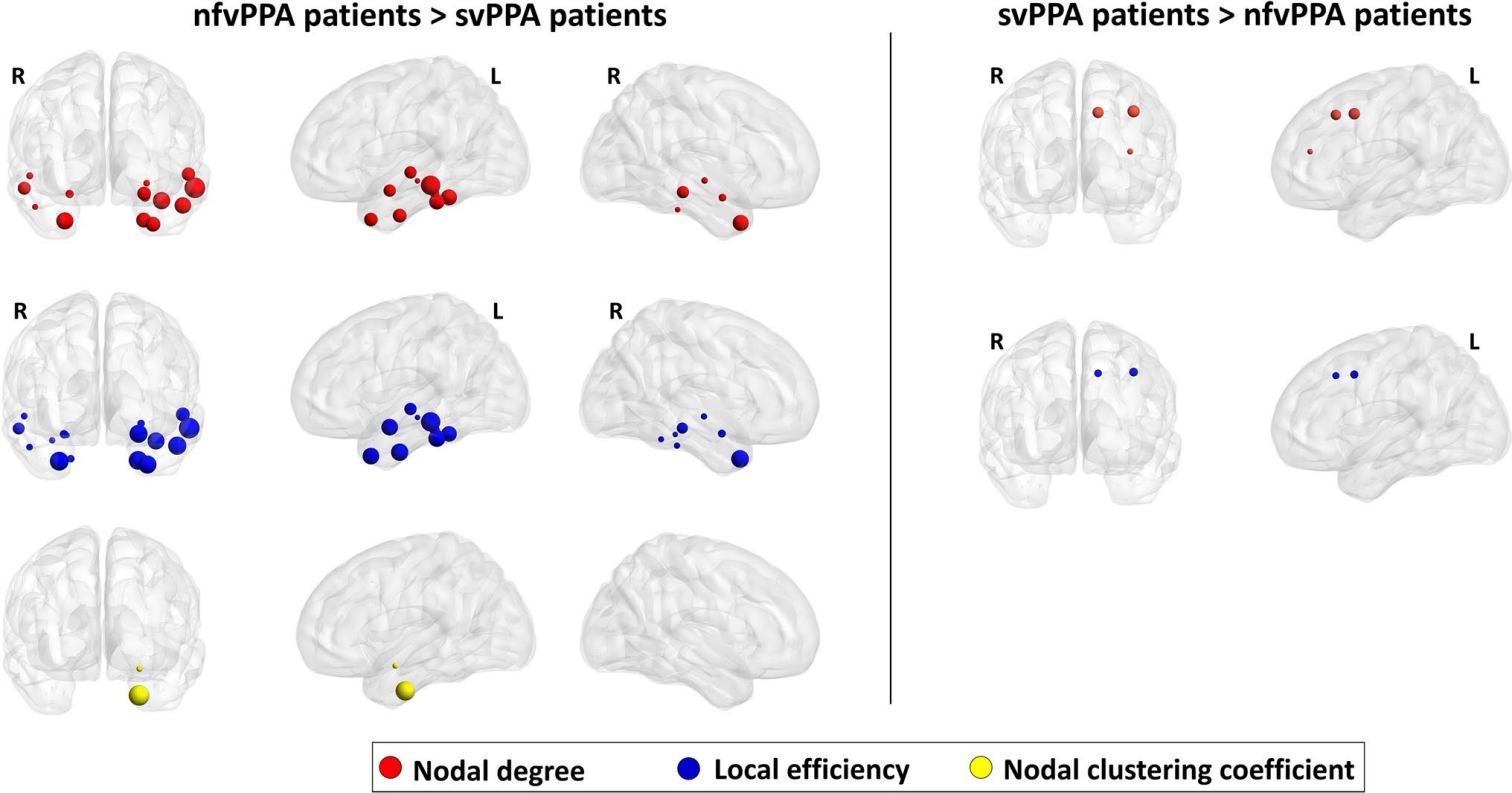
Cortical and subcortical brain regions showing local properties changes between patients with nonfluent variant of primary progressive aphasia (nfvPPA) and patients with semantic variant of primary progressive aphasia (svPPA)(p < 0.05, Bonferroni corrected). Node size is proportional to the effect size of the difference between patient groups

**Fig. 3:**
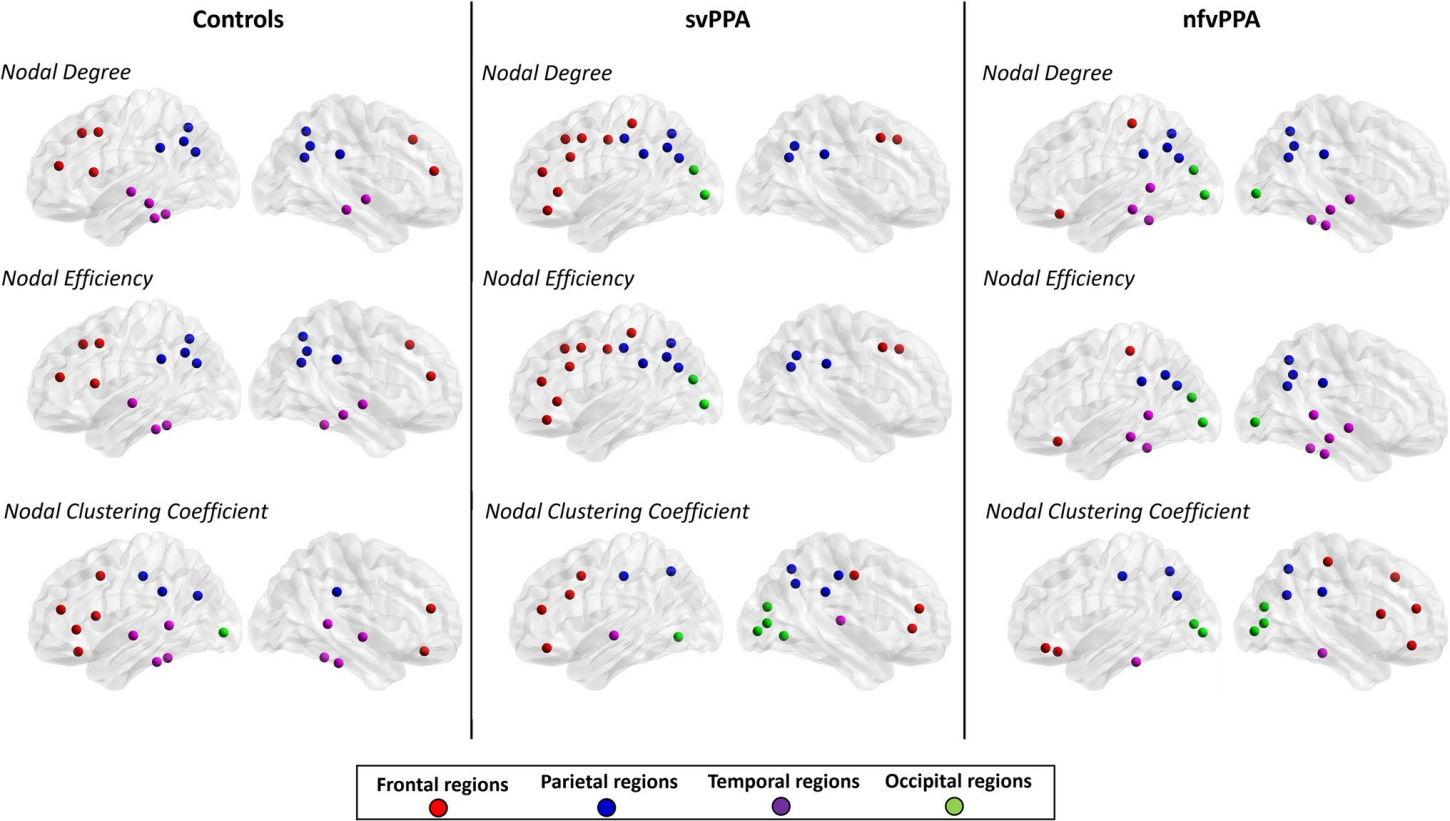
Top 20 ranked brain regions according to local brain measures in controls, patients with semantic variant of primary progressive aphasia (svPPA) and patients with nonfluent variant of primary progressive aphasia (nfvPPA)

Relative to controls, svPPA patients displayed a reduced nodal efficiency, degree and clustering coefficient in several cortical and subcortical brain regions such as the bilateral temporal pole, middle and superiot temporal gyri, entorhinal cortex, amygdala, hippocampus and insula (p-value < 0.04, Bonferroni corrected) (Fig. 1, Table S2). We observed a reduced clustering coefficient, degree and local efficiency in the left caudal middle frontal gyrus, superior frontal gyrus and left pars opercularis of nfvPPA patients compared with controls (p-value < 0.05, Bonferroni corrected) (Fig. 1, Table S3). When local network properties were compared between patient groups, patients with svPPA patients showed decreased local metrics in the temporal pole relative to nfvPPA patients. By contrast, svPPA patients had higher values of nodal degree and nodal efficiency in the left caudal frontal gyrus, left pars opercularis in comparison to nfvPPA patients (Fig. 2, Table S4). Similar differences between PPA groups were observed considering the clinical dementia rating as nuisance variable (Table S5, Supplementary Materials). Compared to control group, ranking of brain regions according to local metric values confirmed network changes in temporal and frontal cortical regions of svPPA and nfvPPA patients, respectively (Fig. 3). No significant correlations were found between clinical variables and local network measures (Table S6, Supplementary Materials).

## Discussion

In the present study, we explored the topological organization of the structural covariance networks in linguistic variants of frontotemporal dementia. Despite the presence of a small-world topology in both patient and control networks, svPPA patients had lower values of normalized clustering coefficient relative to healthy controls and nfvPPA patients. Moreover, svPPA and nfvPPA patients were characterized by higher values of normalized characteristic path length compared with controls. At a local level, patients with svPPA displayed decreased values of local efficiency, clustering coefficient and nodal degree in temporal and limbic brain regions relative to controls and nfvPPA patients. By contrast, local network changes in patients with nfvPPA were focused on frontal brain regions such as the pars opercularis and the caudal middle frontal cortex. Regional network changes between controls and PPA groups were also confirmed by the top-ranked brain regions according to local graph measures. Relative to controls, a loss of ‘importance’ in temporal and frontal brain regions was observed in svPPA and nfvPPA patients, respectively.

Overall, these findings provide new evidences that specific changes in global and local properties characterize the brain network organization of patients with svPPA and nfvPPA. At a global level, higher values of the normalized path length were observed in both svPPA and nfvPPA networks in comparison to control group, suggesting a loss of efficiency in global information communication (Sporns & Zwi, 2004; Watts & Strogatz, 1998). The reduced clustering coefficient found in patients with svPPA is also indicative of a reduced ability in information exchange between close regions (Rubinov & Sporns, 2010; Sporns & Zwi, 2004). These results are in line with previous investigations reporting a reduced integrity in white matter and functional networks of patients with PPA (Mandelli et al., 2018; Ranasinghe et al., 2017; P. A. Reyes et al., 2019). Aberrant network organization was also reported in other dementia syndromes such as the Alzheimer’s disease and behavioral variant of FTD, showing a consistent tendency towards a more randomized architecture compared to healthy controls (Filippi et al., 2013, 2017; Nigro et al., 2021). Global properties changes observed in the current study are thus suggestive of a suboptimal topological organization of the structural covariance networks of svPPA and nfvPPA patients, which may be related to language impairments characterizing both frontotemporal dementia variants.

In support of this idea, svPPA and nfvPPA networks displayed significant local properties abnormalities in specific brain regions that are fundamental to language processing. Relative to controls, patients with svPPA had decreased values of nodal efficiency, clustering coefficient and nodal degree in temporal, entorhinal and fusiform cortex. Moreover, we found reduced local properties in subcortical limbic brain regions such as the amygdala and hippocampus. Structural and functional alterations in the anterior temporal pole have been previously associated with typical verbal and non-verbal semantic impairments observed in svPPA patients (Battistella et al., 2019; Collins et al., 2017; Ding et al., 2020; Guo et al., 2013). Moreover, significant associations were found between the functional connectivity strength of the left anterior hippocampus and performance in semantic tasks (Bocchetta et al., 2019). In particular, naming difficulties were correlated with gray matter atrophy of the anterior temporal pole (Guo et al., 2013). A recent magnetoencephalography study also observed that the damage to the anterior temporal lobe leads to a profound impairments of lexical-semantic system in patients with svPPA. As a compensatory strategy, these patients rely on sublexical processes underpinned by a dorsal route (occipital-parietal) to read irregular words (Borghesani et al., 2020). Our data further demonstrate the critical role of temporal and limbic brain regions in svPPA pathogenesis revealing the existence of a svPPA-related pattern of reduced structural connectivity strength and impaired information processing in these brain regions. These findings are in line with a recent whole brain tractography study revealing a widely altered connectivity network in temporal and occipital regions of svPPA patients (Reyes et al., 2019). Decreased nodal strength in the temporal cortex, left amygdala and hippocampus was also observed in svPPA patients using resting-state functional magnetic resonance imaging and graph analysis (Agosta et al., 2014).

Concerning nfvPPA patients, we observed that local network abnormalities were confined to specific frontal brain regions such as the caudal and superior frontal gyrus and the pars opercularis of the inferior frontal gyrus. A major predominance in local network changes was also observed in the left hemisphere in line with the hypothesis of a selective vulnerability in nfvPPA left brain regions (Leyton et al., 2016; Mandelli et al., 2018; ; Mandelli et al., 2016a, 2016b; Montembeault et al., 2018). Frontal brain regions, together with the dorsal insular, supplementary motor and striatal regions, constitute the speech production network (Mandelli et al., 2016a, 2016b). Damage to this network has been previously associated with fluency and grammatical deficits typically observed in nfvPPA (Mandelli et al., 2018; Mandelli et al., 2016a, 2016b; P. Reyes et al., 2018). Of note, the pars opercularis has been reported to be the earliest region of brain degeneration in nfvPPA patients (Mandelli et al., 2016a, 2016b). Our study further highlights the role of the pars opercularis in nfvPPA revealing long-range and short-range communication deficits between this region and the rest of the brain in patients with nfvPPA when compared to controls.

Of note, differences in local network properties between svPPA and nfvPPA patients are consistent with molecular nexopathy model that proposes a conjunction of pathogenic protein mechanisms and macroanatomical signatures of brain network disintegration (Warren et al., 2012, 2013). Indeed, nfvPPA and svPPA are commonly associated with underlying FTD-tau and TDP pathological aggregates, respectively (Rohrer & Schott, 2011). The distinct patterns of network alterations observed in svPPA and nfvPPA may be thus related to different proteinopathies associated with each PPA syndrome.

The current study has some limitations which have to be pointed out. First, disease duration was not available in data used for this study. Further investigations are therefore required to assess the influence of disease duration on local and global network changes observed in svPPA and nfvPPA patients. Second, patients with nfvPPA showed an older age in comparison to controls and svPPA patients. However, cortical and subcortical morphometric values were corrected by age, sex and total intracranial volume before network construction. Third, no significant correlations were found between network metrics and clinical data in PPA patients. This may suggest that clinical data cannot be solely accounted for by the properties of the underlying structural networks in PPA patients. Therefore, future investigations combining structural and functional network properties may improve the estimation of brain connectivity allowing a better characterization of brain mechanisms underlying different clinical presentations. Moreover, longitudinal studies are required to examine the predictive power of network properties for clinical progression in PPA patients. Finally, gray matter connectivity between brain regions was evaluated after removing the effects of age, gender and TIV on morphometric data. Although this approach allowed to reduce potential confounding effects, no directly investigation of the effect of age and sex on network properties was possible.

## Conclusions

We showed that gray matter covariance analysis might represent a useful tool to investigate the brain network organization in linguistic variants of frontotemporal dementia. Compared to controls, patients with svPPA showed a reduced connectivity and impaired information processing in temporal and limbic brain regions. By contrast, nfvPPA patients were characterized by local network changes in frontal cortical areas. All together, these findings provide further evidence that distinct patterns of structural network alterations are associated with neurodegenerative mechanisms underlying each PPA variant.

## Supplementary Information

Below is the link to the electronic supplementary material.

Supplementary file1 (DOCX 131 kb)

## Data Availability

Data used in current study were collected by the Frontotemporal Lobar Degeneration Neuroimaging Initiative (FTLDNI) (http://memory.ucsf.edu/research) and downloaded through the LONI platform after approved by the data access committee.
