# Supplementary material for: Altered structural brain networks in linguistic variants of frontotemporal dementia
Source: Brain Imaging Behav. 2021 Nov 10;16(3):1113–22. doi: 10.1007/s11682-021-00560-2 (PMC9107413; doi:10.1007/s11682-021-00560-2)
Supplement: Supplementary file 1 — Supplementary file1 (DOCX 131 kb) [file 11682_2021_560_MOESM1_ESM.docx]

**Table S1.** List of cortical and subcortical brain regions used to construct structural covariance networks.

| **Cortical regions** | | | |  |
| --- | --- | --- | --- | --- |
| **Index** | **Name** | **Hemisphere** | **Lobe** | **Label** |
| 1 | Bank of the superior temporal sulcus | L | Temporal | l.bankssts |
| 2 | Caudal anteriocingulate | L | Cingulate | l.caudalanteriorcingulate |
| 3 | Caudal middle frontagyrus | L | Frontal | l.caudalmiddlefrontal |
| 4 | Cuneus | L | Occipital | l.cuneus |
| 5 | Entorhinal | L | Temporal | l.entorhinal |
| 6 | Fusiform | L | Temporal | l.fusiform |
| 7 | Inferior parietal lobule | L | Parietal | l.inferiorparietal |
| 8 | Inferior temporal gyrus | L | Temporal | l.inferiortemporal |
| 9 | Isthmus cingulate cortex | L | Cingulate | l.isthmuscingulate |
| 10 | Lateral occipital gyrus | L | Occipital | l.lateraloccipital |
| 11 | Lateral orbitofrontal | L | Frontal | l.lateralorbitofrontal |
| 12 | Lingual | L | Occipital | l.lingual |
| 13 | Medial orbitofrontal | L | Frontal | l.medialorbitofrontal |
| 14 | Middle temporal gyrus | L | Temporal | l.middletemporal |
| 15 | Parahippocampal | L | Temporal | l.parahippocampal |
| 16 | Paracentral | L | Frontal | l.paracentral |
| 17 | Pars opercularis | L | Frontal | l.parsopercularis |
| 18 | Pars orbitalis | L | Frontal | l.parsorbitalis |
| 19 | Pars triangularis | L | Frontal | l.parstriangularis |
| 20 | Pericalcarine | L | Occipital | l.pericalcarine |
| 21 | Postcentral | L | Parietal | l.postcentral |
| 22 | Posterior cingulate cortex | L | Cingulate | l.posteriorcingulate |
| 23 | Precentral | L | Frontal | l.precentral |
| 24 | Precuneus | L | Parietal | l.precuneus |
| 25 | Rostral anterior cingulate cortex | L | Cingulate | l.rostralanteriorcingulate |
| 26 | Rostral middle frontal gyrus | L | Frontal | l.rostralmiddlefrontal |
| 27 | Superior frontal gyrus | L | Frontal | l.superiorfrontal |
| 28 | Superior parietal lobule | L | Parietal | l.superiorparietal |
| 29 | Superior temporal gyrus | L | Temporal | l.superiortemporal |
| 30 | Supramarginal gyrus | L | Parietal | l.supramarginal |
| 31 | Frontal pole | L | Frontal | l.frontalpole |
| 32 | Temporal pole | L | Temporal | l.temporalpole |
| 33 | Transverse temporal | L | Temporal | l.transversetemporal |
| 34 | Insula | L | Insula | l.insula |
| 35 | Bank of the superior temporal sulcus | R | Temporal | r.bankssts |
| 36 | Caudal anteriocingulate | R | Cingulate | r.caudalanteriorcingulate |
| 37 | Caudal middle frontagyrus | R | Frontal | r.caudalmiddlefrontal |
| 38 | Cuneus | R | Occipital | r.cuneus |
| 39 | Entorhinal | R | Temporal | r.entorhinal |
| 40 | Fusiform | R | Temporal | r.fusiform |
| 41 | Inferior parietal lobule | R | Parietal | r.inferiorparietal |
| 42 | Inferior temporal gyrus | R | Temporal | r.inferiortemporal |
| 43 | Isthmus cingulate cortex | R | Cingulate | r.isthmuscingulate |
| 44 | Lateral occipital gyrus | R | Occipital | r.lateraloccipital |
| 45 | Lateral orbitofrontal | R | Frontal | r.lateralorbitofrontal |
| 46 | Lingual | R | Occipital | r.lingual |
| 47 | Medial orbitofrontal | R | Frontal | r.medialorbitofrontal |
| 48 | Middle temporal gyrus | R | Temporal | r.middletemporal |
| 49 | Parahippocampal | R | Temporal | r.parahippocampal |
| 50 | Paracentral | R | Frontal | r.paracentral |
| 51 | Pars opercularis | R | Frontal | r.parsopercularis |
| 52 | Pars orbitalis | R | Frontal | r.parsorbitalis |
| 53 | Pars triangularis | R | Frontal | r.parstriangularis |
| 54 | Pericalcarine | R | Occipital | r.pericalcarine |
| 55 | Postcentral | R | Parietal | r.postcentral |
| 56 | Posterior cingulate cortex | R | Cingulate | r.posteriorcingulate |
| 57 | Precentral | R | Frontal | r.precentral |
| 58 | Precuneus | R | Parietal | r.precuneus |
| 59 | Rostral anterior cingulate cortex | R | Cingulate | r.rostralanteriorcingulate |
| 60 | Rostral middle frontal gyrus | R | Frontal | r.rostralmiddlefrontal |
| 61 | Superior frontal gyrus | R | Frontal | r.superiorfrontal |
| 62 | Superior parietal lobule | R | Parietal | r.superiorparietal |
| 63 | Superior temporal gyrus | R | Temporal | r.superiortemporal |
| 64 | Supramarginal gyrus | R | Parietal | r.supramarginal |
| 65 | Frontal pole | R | Frontal | r.frontalpole |
| 66 | Temporal pole | R | Temporal | r.temporalpole |
| 67 | Transverse temporal | R | Temporal | r.transversetemporal |
| 68 | Insula | R | Insula | r.insula |
| **Subcortical regions** | | | |  |
| 1 | Thalamus | L | Subcortical | l.thalamus |
| 2 | Hippocampus | L | Subcortical | l.hippocampus |
| 3 | Amygdala | L | Subcortical | l.amygdala |
| 4 | Putamen | L | Subcortical | l.putamen |
| 5 | Pallidum | L | Subcortical | l.pallidum |
| 6 | Caudate | L | Subcortical | l.caudate |
| 7 | Accumbens | L | Subcortical | l.accumbens |
| 8 | Thalamus | R | Subcortical | r.thalamus |
| 9 | Hippocampus | R | Subcortical | r.hippocampus |
| 10 | Amygdala | R | Subcortical | r.amygdala |
| 11 | Putamen | R | Subcortical | r.putamen |
| 12 | Pallidum | R | Subcortical | r.pallidum |
| 13 | Caudate | R | Subcortical | r.caudate |
| 14 | Accumbens | R | Subcortical | r.accumbens |

**Table S2.** Cortical and subcortical brain regions showing nodal degree differences between controls and PPA patients.

| Nodal Degree | | | | | | | | |
| --- | --- | --- | --- | --- | --- | --- | --- | --- |
| HC > svPPA | | | **HC > nfvPPA** | | | **nfvPPA vs svPPA** | | |
| Brain regions | **p-value** | **Effect size** | **Brain regions** | **p-value** | **Effect size** | **Brain regions** | **p-value** | **Effect size** |
| l.inferiortemporal | 0.02 | 2.50 | l.superiorfrontal | 0.02 | 1.43 | l.middletemporal | 0.02 | 2.57 |
| l.superiortemporal | 0.02 | 2.37 | l.caudalmiddlefrontal | 0.02 | 1.39 | l.fusiform | 0.02 | 2.19 |
| l.middletemporal | 0.02 | 2.09 | r.superiorfrontal | 0.02 | 1.00 | r.temporalpole | 0.02 | 2.17 |
| l.fusiform | 0.02 | 2.07 | l.parsopercularis | 0.02 | 0.76 | l.inferiortemporal | 0.02 | 2.10 |
| l.temporalpole | 0.02 | 1.72 |  |  |  | l.entorhinal | 0.02 | 1.92 |
| l.entorhinal | 0.02 | 1.49 |  |  |  | l.temporalpole | 0.02 | 1.90 |
| r.temporalpole | 0.02 | 1.45 |  |  |  | l.amygdala | 0.02 | 1.76 |
| l.amygdala | 0.02 | 1.34 |  |  |  | l.superiortemporal | 0.02 | 1.75 |
| l.hippocampus | 0.02 | 1.29 |  |  |  | r.middletemporal | 0.02 | 1.70 |
| r.middletemporal | 0.02 | 1.19 |  |  |  | l.parahippocampal | 0.02 | 1.40 |
| r.superiortemporal | 0.02 | 1.18 |  |  |  | r.amygdala | 0.02 | 1.15 |
| r.entorhinal | 0.02 | 1.12 |  |  |  | r.superiortemporal | 0.03 | 1.00 |
| r.inferiortemporal | 0.02 | 0.99 |  |  |  | l.hippocampus | 0.03 | 0.96 |
| r.fusiform | 0.02 | 0.92 |  |  |  | r.inferiortemporal | 0.03 | 0.88 |
| l.parahippocampal | 0.02 | 0.89 |  |  |  | l.rostralmiddlefrontal | 0.03 | -0.84 |
| r.amygdala | 0.03 | 0.81 |  |  |  | l.superiorfrontal | 0.02 | -1.46 |
| r.hippocampus | 0.03 | 0.81 |  |  |  | l.caudalmiddlefrontal | 0.02 | -1.58 |
| r.insula | 0.05 | 0.67 |  |  |  |  |  |  |

HC, healthy controls; svPPA, patients with semantic variant of primary progressive aphasia; nfvPPA, patients with non-fluent/agrammatic variant of primary progressive aphasia.

**Table S3.** Cortical and subcortical brain regions showing nodal efficiency differences between controls and PPA patients.

| Nodal Effiiency | | | | | | | | |
| --- | --- | --- | --- | --- | --- | --- | --- | --- |
| HC > svPPA | | | **HC > nfvPPA** | | | **nfvPPA vs svPPA** | | |
| Brain regions | **p-value** | **Effect size** | **Brain regions** | **p-value** | **Effect size** | **Brain regions** | **p-value** | **Effect size** |
| l.inferiortemporal | 0.02 | 3.29 | l.superiorfrontal | 0.02 | 1.51 | l.middletemporal | 0.02 | 2.60 |
| l.superiortemporal | 0.02 | 2.65 | l.caudalmiddlefrontal | 0.02 | 1.50 | r.temporalpole | 0.02 | 2.39 |
| l.fusiform | 0.02 | 2.57 | r.superiorfrontal | 0.02 | 1.13 | l.entorhinal | 0.02 | 2.38 |
| l.middletemporal | 0.02 | 2.43 | l.parsopercularis | 0.02 | 1.05 | l.inferiortemporal | 0.02 | 2.33 |
| l.temporalpole | 0.02 | 2.30 | r.caudalmiddlefrontal | 0.02 | 0.95 | l.temporalpole | 0.02 | 2.31 |
| l.entorhinal | 0.02 | 1.98 | r.parsopercularis | 0.28 | 0.59 | l.amygdala | 0.02 | 2.30 |
| r.temporalpole | 0.02 | 1.95 |  |  |  | l.fusiform | 0.02 | 2.19 |
| l.amygdala | 0.02 | 1.69 |  |  |  | l.superiortemporal | 0.02 | 1.82 |
| l.hippocampus | 0.02 | 1.66 |  |  |  | r.middletemporal | 0.02 | 1.59 |
| r.superiortemporal | 0.02 | 1.57 |  |  |  | l.parahippocampal | 0.02 | 1.58 |
| r.middletemporal | 0.02 | 1.54 |  |  |  | r.amygdala | 0.02 | 1.19 |
| r.entorhinal | 0.02 | 1.36 |  |  |  | l.hippocampus | 0.02 | 1.07 |
| r.inferiortemporal | 0.02 | 1.32 |  |  |  | r.entorhinal | 0.03 | 1.07 |
| r.fusiform | 0.02 | 1.32 |  |  |  | r.superiortemporal | 0.02 | 0.99 |
| l.parahippocampal | 0.02 | 1.08 |  |  |  | r.inferiortemporal | 0.02 | 0.98 |
| r.amygdala | 0.02 | 0.95 |  |  |  | r.fusiform | 0.03 | 0.95 |
| r.hippocampus | 0.02 | 0.91 |  |  |  | r.parahippocampal | 0.03 | 0.89 |
| l.insula | 0.02 | 0.89 |  |  |  | l.superiorfrontal | 0.02 | -1.09 |
| r.insula | 0.03 | 0.84 |  |  |  | l.caudalmiddlefrontal | 0.03 | -1.22 |
| l.bankssts | 0.02 | 0.75 |  |  |  |  |  |  |
| r.rostralmiddlefrontal | 0.03 | 0.69 |  |  |  |  |  |  |

HC, healthy controls; svPPA, patients with semantic variant of primary progressive aphasia; nfvPPA, patients with non-fluent/agrammatic variant of primary progressive aphasia.

**Table S4.** Cortical and subcortical brain regions showing nodal clustering coefficient differences between controls and PPA patients.

| Nodal Clustering coefficient | | | | | | | | |
| --- | --- | --- | --- | --- | --- | --- | --- | --- |
| HC > svPPA | | | **HC > nfvPPA** | | | **nfvPPA > svPPA** | | |
| Brain regions | **p-value** | **Effect size** | **Brain regions** | **p-value** | **Effect size** | **Brain regions** | **p-value** | **Effect size** |
| l.entorhinal | 0.02 | 2.08 | l.caudalmiddlefrontal | 0.02 | 0.85 | l.entorhinal | 0.02 | 1.85 |
| l.hippocampus | 0.02 | 1.33 | l.parstriangularis | 0.02 | 0.74 | l.amygdala | 0.02 | 1.10 |
| l.temporalpole | 0.02 | 1.32 |  |  |  |  |  |  |
| l.middletemporal | 0.02 | 1.01 |  |  |  |  |  |  |
| l.amygdala | 0.02 | 1.00 |  |  |  |  |  |  |
| l.fusiform | 0.02 | 0.98 |  |  |  |  |  |  |
| l.parsopercularis | 0.02 | 0.91 |  |  |  |  |  |  |
| r.inferiortemporal | 0.02 | 0.90 |  |  |  |  |  |  |
| r.bankssts | 0.02 | 0.84 |  |  |  |  |  |  |
| r.superiortemporal | 0.02 | 0.84 |  |  |  |  |  |  |
| l.inferiortemporal | 0.03 | 0.84 |  |  |  |  |  |  |
| r.temporalpole | 0.05 | 0.72 |  |  |  |  |  |  |

HC, healthy controls; svPPA, patients with semantic variant of primary progressive aphasia; nfvPPA, patients with non-fluent/agrammatic variant of primary progressive aphasia.

**Table S5.** Cortical and subcortical brain regions showing local properties differences between PPA patients considering the disease severity as nuisance variable.

| nfvPPA vs svPPA | | | | | | | | |
| --- | --- | --- | --- | --- | --- | --- | --- | --- |
| Nodal Degree | | | **Nodal Efficiency** | | | **Nodal Clustering Coefficient** | | |
| Brain regions | **p-value** | **Effect size** | **Brain regions** | **p-value** | **Effect size** | **Brain regions** | **p-value** | **Effect size** |
| l.middletemporal | 0.02 | 2.15 | l.middletemporal | 0.02 | 2.17 | l.entorhinal | 0.02 | 1.56 |
| r.temporalpole | 0.02 | 2.09 | r.temporalpole | 0.02 | 2.14 | l.amygdala | 0.03 | 1.02 |
| l.fusiform | 0.02 | 1.91 | l.inferiortemporal | 0.02 | 2.02 |  |  |  |
| l.inferiortemporal | 0.02 | 1.86 | l.entorhinal | 0.02 | 1.99 |  |  |  |
| l.temporalpole | 0.02 | 1.65 | l.amygdala | 0.02 | 1.96 |  |  |  |
| l.entorhinal | 0.02 | 1.60 | l.temporalpole | 0.02 | 1.96 |  |  |  |
| l.superiortemporal | 0.02 | 1.52 | l.fusiform | 0.02 | 1.90 |  |  |  |
| l.amygdala | 0.02 | 1.50 | l.superiortemporal | 0.02 | 1.57 |  |  |  |
| r.middletemporal | 0.02 | 1.47 | l.parahippocampal | 0.02 | 1.40 |  |  |  |
| l.parahippocampal | 0.02 | 1.28 | r.middletemporal | 0.02 | 1.36 |  |  |  |
| r.amygdala | 0.03 | 0.94 | r.entorhinal | 0.05 | 0.99 |  |  |  |
| r.superiortemporal | 0.03 | 0.91 | r.amygdala | 0.02 | 0.98 |  |  |  |
| l.caudalmiddlefrontal | 0.02 | -1.65 | l.hippocampus | 0.05 | 0.89 |  |  |  |
| l.superiorfrontal | 0.02 | -1.51 | l.superiorfrontal | 0.02 | -1.28 |  |  |  |
| l.caudalanteriorcingulate | 0.03 | -0.98 | l.caudalmiddlefrontal | 0.02 | -1.37 |  |  |  |
| r.caudalmiddlefrontal | 0.03 | -0.95 |  |  |  |  |  |  |

svPPA, patients with semantic variant of primary progressive aphasia; nfvPPA, patients with non-fluent/agrammatic variant of primary progressive aphasia.

**Table S6.** Correlations between graph metrics and clinical variables in PPA groups.

| **svPPA** | | | | | | | |
| --- | --- | --- | --- | --- | --- | --- | --- |
|  | **Brain region** | **MMSE** | **CDR** | **CDR-SOB** | **Lexical fluency** | **Semantic fluency** | **BNT** |
| Nodal  Degree | r.precentral | 0.50 (0.004) | - | - | - | - | 0.50 (0.004) |
|  |  |  |  |  |  |  |  |
|  |  |  |  |  |  |  |  |
| Nodal Efficiency | l.supramarginal | 0.55 (0.001) | - | -0.50 (0.004) | - | - | - |
|  | r.superiorparietal | 0.50 (0.004) | - | - | - | - | - |
| Nodal Clustering Coefficient | r.hippocampus | - | - | - | - | - | -0.51 (0.003) |
|  | r.parsopercularis | - | -0.51 (0.003) | - | - | - | - |
| **nfvPPA** | | | | | | | |
|  | **Brain region** | **MMSE** | **CDR** | **CDR-SOB** | **Lexical fluency** | **Semantic fluency** | **BNT** |
| Nodal  Degree | l.pericalcarine | - | - | -0.53 (0.002) | - | - | - |
|  | r.isthmuscingulate | - | - | - | - | - | -0.52 (0.003) |
|  |  |  |  |  |  |  |  |
| Nodal Efficiency | l.pericalcarine | - | - | -0.51 (0.004) | - | 0.50 (0.005) | - |
| Nodal Clustering Coeffient | l.caudate | - | -0.51 (0.004) | - | - | - | - |
|  | l.precentral | - | - | - | 0.58 (0.001) | 0.57 (0.001) | - |
|  | r.accumbens | - | - | - | 0.60 (0.001) | 0.60 (0.001) | - |

*Only correlations showing a p-value < 0.005 uncorrected were reported.*

**Table S7.** AUC values for nodal degree in controls and PPA groups

| **Nodal degree** | | | | | | |
| --- | --- | --- | --- | --- | --- | --- |
|  | **Controls** | | **svPPA** | | **nfvPPA** | |
| **Brain region** | **Mean** | **STD** | **Mean** | **STD** | **Mean** | **STD** |
| l.bankssts | 6.51 | 2.38 | 5.20 | 2.57 | 6.48 | 2.13 |
| l.caudalanteriorcingulate | 5.37 | 2.68 | 7.18 | 1.96 | 4.89 | 2.72 |
| l.caudalmiddlefrontal | 7.08 | 2.13 | 7.63 | 1.66 | 3.81 | 2.99 |
| l.cuneus | 5.42 | 2.50 | 7.21 | 1.88 | 6.55 | 2.09 |
| l.entorhinal | 4.41 | 2.91 | 0.56 | 0.87 | 4.38 | 2.68 |
| l.fusiform | 6.93 | 1.98 | 2.86 | 1.91 | 6.90 | 1.78 |
| l.inferiorparietal | 7.44 | 1.83 | 7.26 | 1.70 | 7.39 | 1.38 |
| l.inferiortemporal | 6.78 | 1.92 | 2.01 | 1.83 | 6.35 | 2.26 |
| l.isthmuscingulate | 5.56 | 2.72 | 6.07 | 2.38 | 5.44 | 2.88 |
| l.lateraloccipital | 6.76 | 2.13 | 7.53 | 2.00 | 6.59 | 2.14 |
| l.lateralorbitofrontal | 6.73 | 2.24 | 6.14 | 2.10 | 6.61 | 2.28 |
| l.lingual | 6.30 | 2.51 | 6.84 | 2.23 | 6.43 | 2.11 |
| l.medialorbitofrontal | 6.28 | 2.44 | 5.51 | 2.27 | 6.06 | 2.38 |
| l.middletemporal | 6.83 | 2.28 | 2.30 | 1.74 | 6.87 | 1.82 |
| l.parahippocampal | 4.86 | 2.81 | 2.53 | 1.77 | 5.54 | 2.48 |
| l.paracentral | 6.42 | 2.64 | 7.49 | 1.80 | 6.59 | 2.21 |
| l.parsopercularis | 6.86 | 1.90 | 7.02 | 1.90 | 5.16 | 3.11 |
| l.parsorbitalis | 6.57 | 2.46 | 7.24 | 1.83 | 5.72 | 2.54 |
| l.parstriangularis | 6.40 | 2.35 | 7.41 | 1.71 | 5.65 | 2.51 |
| l.pericalcarine | 5.39 | 2.71 | 5.86 | 3.02 | 5.82 | 2.53 |
| l.postcentral | 6.56 | 2.28 | 7.32 | 1.90 | 6.23 | 2.15 |
| l.posteriorcingulate | 5.72 | 2.52 | 6.70 | 2.30 | 6.15 | 2.38 |
| l.precentral | 6.70 | 2.19 | 7.38 | 1.85 | 5.83 | 2.56 |
| l.precuneus | 7.03 | 2.02 | 7.89 | 1.46 | 6.80 | 2.12 |
| l.rostralanteriorcingulate | 5.83 | 2.59 | 6.07 | 2.37 | 5.24 | 2.72 |
| l.rostralmiddlefrontal | 6.94 | 2.11 | 7.91 | 1.36 | 6.31 | 2.33 |
| l.superiorfrontal | 7.34 | 1.83 | 7.47 | 1.47 | 4.45 | 2.54 |
| l.superiorparietal | 7.08 | 2.10 | 7.59 | 1.81 | 6.48 | 2.27 |
| l.superiortemporal | 7.14 | 1.87 | 2.78 | 1.77 | 6.43 | 2.37 |
| l.supramarginal | 7.62 | 1.53 | 7.71 | 1.70 | 7.45 | 1.40 |
| l.frontalpole | 5.42 | 2.88 | 6.26 | 2.39 | 5.17 | 2.93 |
| l.temporalpole | 5.36 | 2.88 | 0.96 | 0.86 | 4.34 | 2.36 |
| l.transversetemporal | 5.17 | 2.96 | 5.09 | 2.54 | 5.49 | 2.51 |
| l.insula | 6.11 | 2.53 | 4.38 | 2.55 | 6.07 | 2.29 |
| r.bankssts | 6.05 | 2.46 | 5.66 | 2.90 | 6.39 | 2.03 |
| r.caudalanteriorcingulate | 5.10 | 2.88 | 5.89 | 2.53 | 5.91 | 2.44 |
| r.caudalmiddlefrontal | 6.72 | 2.08 | 7.32 | 1.83 | 5.10 | 2.97 |
| r.cuneus | 5.56 | 2.82 | 6.12 | 2.79 | 6.05 | 2.37 |
| r.entorhinal | 4.80 | 2.74 | 1.87 | 2.19 | 3.94 | 2.69 |
| r.fusiform | 6.75 | 2.18 | 4.69 | 2.38 | 6.65 | 2.29 |
| r.inferiorparietal | 7.32 | 1.87 | 7.28 | 2.02 | 7.54 | 1.17 |
| r.inferiortemporal | 6.71 | 2.12 | 4.50 | 2.57 | 6.69 | 2.39 |
| r.isthmuscingulate | 5.20 | 2.86 | 5.92 | 2.39 | 5.91 | 2.39 |
| r.lateraloccipital | 6.65 | 2.06 | 7.06 | 1.98 | 7.04 | 1.93 |
| r.lateralorbitofrontal | 6.04 | 2.51 | 6.04 | 2.68 | 6.19 | 2.62 |
| r.lingual | 6.41 | 2.39 | 6.63 | 2.32 | 6.38 | 2.18 |
| r.medialorbitofrontal | 6.04 | 2.46 | 6.93 | 2.41 | 5.84 | 2.57 |
| r.middletemporal | 6.81 | 1.92 | 4.46 | 2.16 | 7.60 | 1.46 |
| r.parahippocampal | 5.00 | 2.79 | 4.28 | 2.27 | 5.92 | 2.32 |
| r.paracentral | 6.36 | 2.46 | 5.75 | 2.98 | 6.27 | 2.08 |
| r.parsopercularis | 6.61 | 2.15 | 6.68 | 2.40 | 5.59 | 2.29 |
| r.parsorbitalis | 6.34 | 2.55 | 6.81 | 2.40 | 5.91 | 2.33 |
| r.parstriangularis | 6.70 | 2.10 | 6.61 | 2.46 | 6.09 | 2.59 |
| r.pericalcarine | 5.34 | 2.66 | 6.64 | 2.65 | 5.66 | 2.06 |
| r.postcentral | 6.51 | 2.23 | 6.82 | 2.41 | 6.33 | 2.27 |
| r.posteriorcingulate | 5.78 | 2.68 | 7.00 | 2.07 | 6.06 | 2.29 |
| r.precentral | 6.64 | 2.17 | 7.06 | 1.91 | 6.23 | 2.71 |
| r.precuneus | 7.01 | 2.17 | 7.43 | 1.47 | 7.48 | 1.44 |
| r.rostralanteriorcingulate | 5.69 | 2.63 | 6.91 | 2.24 | 5.71 | 2.65 |
| r.rostralmiddlefrontal | 7.04 | 1.90 | 6.60 | 1.90 | 6.45 | 1.96 |
| r.superiorfrontal | 7.38 | 1.73 | 7.37 | 2.10 | 5.44 | 2.55 |
| r.superiorparietal | 7.03 | 2.08 | 7.11 | 2.06 | 7.08 | 2.13 |
| r.superiortemporal | 7.09 | 1.93 | 4.65 | 2.46 | 6.95 | 2.14 |
| r.supramarginal | 7.14 | 1.83 | 7.24 | 1.88 | 7.13 | 1.79 |
| r.frontalpole | 4.56 | 2.67 | 6.31 | 2.41 | 5.32 | 3.14 |
| r.temporalpole | 5.06 | 2.93 | 1.26 | 0.97 | 5.41 | 2.52 |
| r.transversetemporal | 5.29 | 2.90 | 5.81 | 2.41 | 5.24 | 2.60 |
| r.insula | 6.55 | 2.28 | 4.99 | 2.37 | 6.41 | 2.11 |
| l.thalamus | 4.38 | 3.00 | 3.78 | 2.39 | 3.78 | 2.73 |
| l.hippocampus | 3.55 | 2.71 | 4.57 | 2.73 | 3.47 | 3.15 |
| l.amygdala | 4.24 | 2.96 | 2.72 | 2.28 | 3.79 | 2.85 |
| l.putamen | 3.83 | 2.75 | 5.11 | 2.45 | 4.01 | 2.76 |
| l.pallidum | 4.57 | 2.89 | 1.19 | 1.39 | 3.33 | 2.81 |
| l.caudate | 4.49 | 3.09 | 0.82 | 0.89 | 4.69 | 2.98 |
| l.accumbens | 3.52 | 2.72 | 2.52 | 2.43 | 3.96 | 3.00 |
| r.thalamus | 4.28 | 3.08 | 5.03 | 2.63 | 4.85 | 2.74 |
| r.hippocampus | 3.46 | 2.94 | 4.83 | 2.85 | 3.09 | 2.72 |
| r.amygdala | 4.15 | 2.92 | 3.73 | 2.65 | 4.10 | 3.13 |
| r.putamen | 3.71 | 2.87 | 4.84 | 2.88 | 4.53 | 2.74 |
| r.pallidum | 4.87 | 2.90 | 2.64 | 2.19 | 3.67 | 2.71 |
| r.caudate | 4.24 | 3.06 | 1.96 | 1.62 | 4.62 | 2.84 |
| r.accumbens | 4.01 | 3.01 | 4.91 | 3.11 | 4.27 | 2.82 |

**Table S8.** AUC values for nodal efficiency in controls and PPA groups.

| **Nodal efficiency** | | | | | | |
| --- | --- | --- | --- | --- | --- | --- |
|  | **Controls** | | **svPPA** | | **nfvPPA** | |
| **Brain region** | **Mean** | **Std** | **Mean** | **Std** | **Mean** | **Std** |
| l.bankssts | 0.14 | 0.03 | 0.11 | 0.04 | 0.14 | 0.03 |
| l.caudalanteriorcingulate | 0.12 | 0.04 | 0.14 | 0.02 | 0.11 | 0.04 |
| l.caudalmiddlefrontal | 0.15 | 0.03 | 0.14 | 0.02 | 0.09 | 0.06 |
| l.cuneus | 0.13 | 0.04 | 0.14 | 0.02 | 0.14 | 0.03 |
| l.entorhinal | 0.11 | 0.05 | 0.02 | 0.03 | 0.11 | 0.05 |
| l.fusiform | 0.15 | 0.02 | 0.08 | 0.04 | 0.14 | 0.02 |
| l.inferiorparietal | 0.15 | 0.02 | 0.14 | 0.02 | 0.15 | 0.02 |
| l.inferiortemporal | 0.14 | 0.02 | 0.06 | 0.03 | 0.13 | 0.03 |
| l.isthmuscingulate | 0.13 | 0.04 | 0.13 | 0.03 | 0.12 | 0.04 |
| l.lateraloccipital | 0.14 | 0.03 | 0.14 | 0.02 | 0.14 | 0.03 |
| l.lateralorbitofrontal | 0.14 | 0.03 | 0.13 | 0.02 | 0.14 | 0.03 |
| l.lingual | 0.14 | 0.03 | 0.13 | 0.02 | 0.14 | 0.03 |
| l.medialorbitofrontal | 0.14 | 0.03 | 0.12 | 0.03 | 0.13 | 0.03 |
| l.middletemporal | 0.14 | 0.03 | 0.06 | 0.04 | 0.14 | 0.02 |
| l.parahippocampal | 0.12 | 0.04 | 0.07 | 0.03 | 0.12 | 0.04 |
| l.paracentral | 0.14 | 0.04 | 0.14 | 0.02 | 0.14 | 0.03 |
| l.parsopercularis | 0.15 | 0.02 | 0.14 | 0.02 | 0.11 | 0.05 |
| l.parsorbitalis | 0.14 | 0.03 | 0.14 | 0.02 | 0.13 | 0.04 |
| l.parstriangularis | 0.14 | 0.03 | 0.14 | 0.02 | 0.13 | 0.04 |
| l.pericalcarine | 0.12 | 0.04 | 0.12 | 0.04 | 0.13 | 0.03 |
| l.postcentral | 0.14 | 0.03 | 0.14 | 0.02 | 0.13 | 0.03 |
| l.posteriorcingulate | 0.13 | 0.03 | 0.13 | 0.03 | 0.13 | 0.03 |
| l.precentral | 0.14 | 0.03 | 0.14 | 0.02 | 0.13 | 0.04 |
| l.precuneus | 0.15 | 0.02 | 0.15 | 0.02 | 0.14 | 0.03 |
| l.rostralanteriorcingulate | 0.13 | 0.03 | 0.13 | 0.03 | 0.12 | 0.04 |
| l.rostralmiddlefrontal | 0.15 | 0.03 | 0.15 | 0.01 | 0.13 | 0.03 |
| l.superiorfrontal | 0.15 | 0.02 | 0.14 | 0.01 | 0.11 | 0.04 |
| l.superiorparietal | 0.15 | 0.03 | 0.14 | 0.02 | 0.14 | 0.03 |
| l.superiortemporal | 0.15 | 0.02 | 0.08 | 0.03 | 0.14 | 0.03 |
| l.supramarginal | 0.15 | 0.02 | 0.14 | 0.02 | 0.15 | 0.02 |
| l.frontalpole | 0.12 | 0.05 | 0.13 | 0.03 | 0.12 | 0.05 |
| l.temporalpole | 0.12 | 0.04 | 0.03 | 0.03 | 0.11 | 0.04 |
| l.transversetemporal | 0.12 | 0.05 | 0.11 | 0.03 | 0.12 | 0.03 |
| l.insula | 0.13 | 0.03 | 0.10 | 0.04 | 0.13 | 0.03 |
| r.bankssts | 0.13 | 0.03 | 0.12 | 0.04 | 0.14 | 0.02 |
| r.caudalanteriorcingulate | 0.12 | 0.04 | 0.12 | 0.03 | 0.13 | 0.04 |
| r.caudalmiddlefrontal | 0.14 | 0.03 | 0.14 | 0.02 | 0.11 | 0.05 |
| r.cuneus | 0.13 | 0.04 | 0.12 | 0.03 | 0.13 | 0.03 |
| r.entorhinal | 0.11 | 0.05 | 0.05 | 0.04 | 0.10 | 0.05 |
| r.fusiform | 0.14 | 0.03 | 0.11 | 0.04 | 0.14 | 0.03 |
| r.inferiorparietal | 0.15 | 0.02 | 0.14 | 0.02 | 0.15 | 0.01 |
| r.inferiortemporal | 0.14 | 0.03 | 0.10 | 0.04 | 0.14 | 0.03 |
| r.isthmuscingulate | 0.12 | 0.04 | 0.12 | 0.03 | 0.13 | 0.03 |
| r.lateraloccipital | 0.14 | 0.02 | 0.14 | 0.02 | 0.14 | 0.02 |
| r.lateralorbitofrontal | 0.13 | 0.03 | 0.12 | 0.03 | 0.13 | 0.04 |
| r.lingual | 0.14 | 0.03 | 0.13 | 0.03 | 0.14 | 0.03 |
| r.medialorbitofrontal | 0.13 | 0.03 | 0.13 | 0.03 | 0.13 | 0.03 |
| r.middletemporal | 0.14 | 0.02 | 0.10 | 0.04 | 0.15 | 0.02 |
| r.parahippocampal | 0.12 | 0.04 | 0.10 | 0.04 | 0.13 | 0.03 |
| r.paracentral | 0.14 | 0.03 | 0.12 | 0.04 | 0.13 | 0.03 |
| r.parsopercularis | 0.14 | 0.03 | 0.13 | 0.03 | 0.13 | 0.03 |
| r.parsorbitalis | 0.14 | 0.03 | 0.13 | 0.03 | 0.13 | 0.03 |
| r.parstriangularis | 0.14 | 0.03 | 0.13 | 0.03 | 0.13 | 0.03 |
| r.pericalcarine | 0.12 | 0.04 | 0.13 | 0.03 | 0.13 | 0.03 |
| r.postcentral | 0.14 | 0.03 | 0.13 | 0.03 | 0.13 | 0.03 |
| r.posteriorcingulate | 0.13 | 0.04 | 0.14 | 0.03 | 0.13 | 0.03 |
| r.precentral | 0.14 | 0.03 | 0.14 | 0.02 | 0.13 | 0.04 |
| r.precuneus | 0.15 | 0.02 | 0.14 | 0.01 | 0.15 | 0.02 |
| r.rostralanteriorcingulate | 0.13 | 0.04 | 0.14 | 0.03 | 0.12 | 0.04 |
| r.rostralmiddlefrontal | 0.15 | 0.02 | 0.13 | 0.02 | 0.14 | 0.02 |
| r.superiorfrontal | 0.15 | 0.02 | 0.14 | 0.02 | 0.12 | 0.04 |
| r.superiorparietal | 0.15 | 0.02 | 0.14 | 0.02 | 0.14 | 0.03 |
| r.superiortemporal | 0.15 | 0.02 | 0.11 | 0.04 | 0.14 | 0.03 |
| r.supramarginal | 0.15 | 0.02 | 0.14 | 0.02 | 0.14 | 0.02 |
| r.frontalpole | 0.11 | 0.05 | 0.13 | 0.03 | 0.11 | 0.05 |
| r.temporalpole | 0.12 | 0.04 | 0.04 | 0.03 | 0.12 | 0.04 |
| r.transversetemporal | 0.12 | 0.04 | 0.12 | 0.03 | 0.12 | 0.04 |
| r.insula | 0.14 | 0.03 | 0.11 | 0.03 | 0.14 | 0.02 |
| l.thalamus | 0.11 | 0.05 | 0.09 | 0.03 | 0.10 | 0.04 |
| l.hippocampus | 0.09 | 0.05 | 0.10 | 0.04 | 0.08 | 0.06 |
| l.amygdala | 0.10 | 0.05 | 0.07 | 0.04 | 0.09 | 0.05 |
| l.putamen | 0.10 | 0.05 | 0.11 | 0.03 | 0.10 | 0.05 |
| l.pallidum | 0.11 | 0.05 | 0.04 | 0.03 | 0.08 | 0.05 |
| l.caudate | 0.11 | 0.05 | 0.02 | 0.03 | 0.11 | 0.05 |
| l.accumbens | 0.09 | 0.05 | 0.07 | 0.04 | 0.10 | 0.05 |
| r.thalamus | 0.10 | 0.05 | 0.11 | 0.04 | 0.11 | 0.04 |
| r.hippocampus | 0.09 | 0.05 | 0.11 | 0.04 | 0.08 | 0.05 |
| r.amygdala | 0.10 | 0.05 | 0.09 | 0.05 | 0.10 | 0.06 |
| r.putamen | 0.09 | 0.05 | 0.11 | 0.04 | 0.11 | 0.05 |
| r.pallidum | 0.11 | 0.05 | 0.07 | 0.04 | 0.09 | 0.05 |
| r.caudate | 0.10 | 0.05 | 0.06 | 0.04 | 0.11 | 0.05 |
| r.accumbens | 0.10 | 0.05 | 0.11 | 0.04 | 0.10 | 0.04 |

**Table S9.** AUC values for nodal clustering coefficient in controls and PPA groups.

| **Nodal Clustering Coefficient** | | | | | | |
| --- | --- | --- | --- | --- | --- | --- |
|  | **Controls** | | **svPPA** | | **nfvPPA** | |
| **Brain region** | **Mean** | **Std** | **Mean** | **Std** | **Mean** | **Std** |
| l.bankssts | 0.23 | 0.03 | 0.21 | 0.04 | 0.21 | 0.03 |
| l.caudalanteriorcingulate | 0.22 | 0.05 | 0.22 | 0.02 | 0.22 | 0.03 |
| l.caudalmiddlefrontal | 0.23 | 0.02 | 0.22 | 0.02 | 0.19 | 0.08 |
| l.cuneus | 0.22 | 0.04 | 0.22 | 0.02 | 0.22 | 0.02 |
| l.entorhinal | 0.21 | 0.06 | 0.06 | 0.09 | 0.20 | 0.06 |
| l.fusiform | 0.23 | 0.02 | 0.20 | 0.04 | 0.22 | 0.02 |
| l.inferiorparietal | 0.23 | 0.01 | 0.22 | 0.02 | 0.22 | 0.01 |
| l.inferiortemporal | 0.23 | 0.02 | 0.20 | 0.07 | 0.22 | 0.02 |
| l.isthmuscingulate | 0.22 | 0.04 | 0.21 | 0.02 | 0.21 | 0.05 |
| l.lateraloccipital | 0.23 | 0.02 | 0.22 | 0.02 | 0.22 | 0.01 |
| l.lateralorbitofrontal | 0.23 | 0.02 | 0.21 | 0.02 | 0.22 | 0.02 |
| l.lingual | 0.22 | 0.03 | 0.22 | 0.02 | 0.22 | 0.03 |
| l.medialorbitofrontal | 0.22 | 0.03 | 0.21 | 0.02 | 0.22 | 0.02 |
| l.middletemporal | 0.22 | 0.04 | 0.18 | 0.06 | 0.22 | 0.01 |
| l.parahippocampal | 0.22 | 0.05 | 0.21 | 0.05 | 0.21 | 0.04 |
| l.paracentral | 0.22 | 0.04 | 0.22 | 0.02 | 0.22 | 0.02 |
| l.parsopercularis | 0.23 | 0.02 | 0.21 | 0.02 | 0.22 | 0.03 |
| l.parsorbitalis | 0.22 | 0.03 | 0.22 | 0.01 | 0.22 | 0.03 |
| l.parstriangularis | 0.23 | 0.02 | 0.22 | 0.02 | 0.21 | 0.04 |
| l.pericalcarine | 0.21 | 0.05 | 0.21 | 0.03 | 0.22 | 0.02 |
| l.postcentral | 0.23 | 0.02 | 0.22 | 0.02 | 0.23 | 0.02 |
| l.posteriorcingulate | 0.22 | 0.04 | 0.21 | 0.03 | 0.21 | 0.04 |
| l.precentral | 0.22 | 0.03 | 0.22 | 0.02 | 0.22 | 0.02 |
| l.precuneus | 0.22 | 0.02 | 0.22 | 0.02 | 0.21 | 0.03 |
| l.rostralanteriorcingulate | 0.22 | 0.03 | 0.21 | 0.02 | 0.21 | 0.04 |
| l.rostralmiddlefrontal | 0.23 | 0.02 | 0.22 | 0.02 | 0.22 | 0.03 |
| l.superiorfrontal | 0.23 | 0.02 | 0.22 | 0.02 | 0.20 | 0.05 |
| l.superiorparietal | 0.22 | 0.02 | 0.22 | 0.02 | 0.22 | 0.02 |
| l.superiortemporal | 0.23 | 0.02 | 0.22 | 0.03 | 0.22 | 0.03 |
| l.supramarginal | 0.23 | 0.02 | 0.22 | 0.01 | 0.22 | 0.02 |
| l.frontalpole | 0.21 | 0.05 | 0.21 | 0.04 | 0.21 | 0.06 |
| l.temporalpole | 0.21 | 0.05 | 0.13 | 0.09 | 0.20 | 0.07 |
| l.transversetemporal | 0.21 | 0.06 | 0.21 | 0.03 | 0.22 | 0.04 |
| l.insula | 0.22 | 0.03 | 0.21 | 0.03 | 0.22 | 0.02 |
| r.bankssts | 0.23 | 0.02 | 0.21 | 0.05 | 0.22 | 0.02 |
| r.caudalanteriorcingulate | 0.22 | 0.04 | 0.22 | 0.02 | 0.22 | 0.03 |
| r.caudalmiddlefrontal | 0.22 | 0.02 | 0.22 | 0.02 | 0.22 | 0.05 |
| r.cuneus | 0.22 | 0.05 | 0.22 | 0.04 | 0.23 | 0.02 |
| r.entorhinal | 0.21 | 0.07 | 0.16 | 0.08 | 0.20 | 0.07 |
| r.fusiform | 0.23 | 0.02 | 0.22 | 0.03 | 0.22 | 0.02 |
| r.inferiorparietal | 0.23 | 0.02 | 0.22 | 0.02 | 0.22 | 0.01 |
| r.inferiortemporal | 0.23 | 0.02 | 0.20 | 0.04 | 0.22 | 0.02 |
| r.isthmuscingulate | 0.22 | 0.04 | 0.21 | 0.03 | 0.21 | 0.03 |
| r.lateraloccipital | 0.22 | 0.02 | 0.22 | 0.02 | 0.22 | 0.02 |
| r.lateralorbitofrontal | 0.23 | 0.02 | 0.21 | 0.02 | 0.21 | 0.05 |
| r.lingual | 0.23 | 0.02 | 0.22 | 0.02 | 0.22 | 0.02 |
| r.medialorbitofrontal | 0.23 | 0.03 | 0.21 | 0.04 | 0.22 | 0.03 |
| r.middletemporal | 0.22 | 0.03 | 0.22 | 0.02 | 0.22 | 0.01 |
| r.parahippocampal | 0.22 | 0.05 | 0.21 | 0.03 | 0.22 | 0.02 |
| r.paracentral | 0.23 | 0.02 | 0.22 | 0.05 | 0.22 | 0.02 |
| r.parsopercularis | 0.22 | 0.02 | 0.22 | 0.02 | 0.22 | 0.03 |
| r.parsorbitalis | 0.22 | 0.04 | 0.22 | 0.03 | 0.23 | 0.02 |
| r.parstriangularis | 0.23 | 0.02 | 0.21 | 0.03 | 0.22 | 0.02 |
| r.pericalcarine | 0.22 | 0.04 | 0.22 | 0.02 | 0.23 | 0.02 |
| r.postcentral | 0.22 | 0.04 | 0.22 | 0.02 | 0.22 | 0.02 |
| r.posteriorcingulate | 0.22 | 0.05 | 0.21 | 0.03 | 0.21 | 0.03 |
| r.precentral | 0.22 | 0.03 | 0.22 | 0.02 | 0.20 | 0.06 |
| r.precuneus | 0.22 | 0.02 | 0.22 | 0.01 | 0.22 | 0.01 |
| r.rostralanteriorcingulate | 0.22 | 0.04 | 0.22 | 0.02 | 0.21 | 0.04 |
| r.rostralmiddlefrontal | 0.23 | 0.02 | 0.22 | 0.01 | 0.23 | 0.02 |
| r.superiorfrontal | 0.23 | 0.01 | 0.22 | 0.01 | 0.22 | 0.03 |
| r.superiorparietal | 0.22 | 0.02 | 0.22 | 0.02 | 0.22 | 0.02 |
| r.superiortemporal | 0.23 | 0.02 | 0.21 | 0.03 | 0.22 | 0.02 |
| r.supramarginal | 0.23 | 0.02 | 0.22 | 0.02 | 0.23 | 0.02 |
| r.frontalpole | 0.21 | 0.06 | 0.22 | 0.02 | 0.20 | 0.07 |
| r.temporalpole | 0.22 | 0.04 | 0.18 | 0.09 | 0.21 | 0.04 |
| r.transversetemporal | 0.21 | 0.06 | 0.22 | 0.02 | 0.21 | 0.04 |
| r.insula | 0.22 | 0.03 | 0.20 | 0.03 | 0.22 | 0.01 |
| l.thalamus | 0.21 | 0.06 | 0.21 | 0.05 | 0.20 | 0.06 |
| l.hippocampus | 0.20 | 0.07 | 0.20 | 0.05 | 0.20 | 0.07 |
| l.amygdala | 0.20 | 0.07 | 0.19 | 0.05 | 0.21 | 0.06 |
| l.putamen | 0.20 | 0.07 | 0.20 | 0.04 | 0.19 | 0.06 |
| l.pallidum | 0.21 | 0.06 | 0.12 | 0.10 | 0.19 | 0.08 |
| l.caudate | 0.19 | 0.08 | 0.11 | 0.10 | 0.20 | 0.06 |
| l.accumbens | 0.19 | 0.08 | 0.18 | 0.06 | 0.20 | 0.07 |
| r.thalamus | 0.21 | 0.06 | 0.20 | 0.05 | 0.21 | 0.06 |
| r.hippocampus | 0.19 | 0.08 | 0.21 | 0.04 | 0.20 | 0.07 |
| r.amygdala | 0.21 | 0.06 | 0.20 | 0.06 | 0.19 | 0.07 |
| r.putamen | 0.20 | 0.07 | 0.20 | 0.04 | 0.21 | 0.06 |
| r.pallidum | 0.22 | 0.05 | 0.18 | 0.07 | 0.18 | 0.07 |
| r.caudate | 0.20 | 0.07 | 0.19 | 0.07 | 0.20 | 0.05 |
| r.accumbens | 0.20 | 0.08 | 0.21 | 0.04 | 0.21 | 0.05 |
